# Study of Nonlinear Excitation Circuits for Fluxgate Magnetometer

**DOI:** 10.3390/s23052618

**Published:** 2023-02-27

**Authors:** Chenhao Zhang, Yiming Zhang, Xuhong Wang, Hongyan Meng

**Affiliations:** Faculty of Information Technology, Beijing University of Technology, Beijing 100124, China

**Keywords:** fluxgate, nonlinear, simulation, excitation

## Abstract

This paper presents the common methods and corresponding drawbacks concerning nonlinear analysis of fluxgate excitation circuits and emphasizes the importance of nonlinear analysis for these circuits. With regard to the nonlinearity of the excitation circuit, this paper proposes the use of the core-measured hysteresis curve for mathematical analysis and the use of a nonlinear model that considers the coupling effect of the core and winding and influence of the historical magnetic field on the core for simulation analysis. The feasibility of mathematical calculations and simulation for the nonlinear study of fluxgate excitation circuit is verified via experiments. The results demonstrate that, in this regard, the simulation is four times better than a mathematical calculation. The simulation and experimental results of the excitation current and voltage waveforms under different excitation circuit parameters and structures are essentially consistent, with a difference in current of no more than 1 mA, thereby verifying the effectiveness of the nonlinear excitation analysis method.

## 1. Introduction

Fluxgate sensors measure weak static magnetic fields ranging between 0.1 and 10,000 nT. The earliest studies on fluxgate sensors dates to the early 1930s, when an iron core was the core material used for fluxgate and closed-loop feedback was not used, which limited their accuracy, linearity, and other performance indicators [1]. With the continuous development of magnetic materials and electronic technology, most materials currently used in fluxgates are soft magnetic materials, such as permalloy and amorphous, which have the characteristics of low coercivity and high permeability. These materials enable the core to achieve high sensitivity, low noise, and low excitation power [2]. Additionally, the components used in the entire fluxgate circuit part are more integrated, more free of noise, and have an algorithm to improve the overall linearity with feedback [3]. Modern fluxgate sensors can achieve 1 pT noise and 0.01 nT resolution [4]. Fluxgate sensors have been widely used in geological exploration, geomagnetic field observations, and magnetic measurements in space since their invention because of their simple structure, low cost, and high reliability [5].

The core magnetic material, excitation circuit topology, and component parameters all play a significant role in the fluxgate performance. The core material and excitation circuit have been studied throughout the development of fluxgates [6]. Early magnetic core materials were ferromagnetic with high coercivity and large hysteresis areas, which resulted in high excitation power loss and noise. The core materials were developed into soft magnetic materials using a significantly lower coercivity and higher permeability, and the excitation current required for the core to enter deep saturation was significantly reduced. Subsequently, significant developments were made in the process and structure of the core materials, which were annealed at specific temperatures to improve their magnetic properties further [7]. The magnetic properties of core materials can be enhanced by annealing at specific temperatures, and the thin strip toroidal core structure is mostly used [8]. Further optimization of the coil significantly minimizes the magnetic noise and bias, increases magnetic permeability, and reduces the interference of the excitation field with the induction signal passing through the structure [9,10]. The sinusoidal signal that the signal generator produced was initially directly connected to the excitation coil as the excitation waveform in the excitation circuit. The average power consumption of the circuit was high, and its overall structure was complex and unportable [11]. As digital circuits have advanced, the excitation signal is now driven using a chip and then passed into the excitation circuit. However, only one capacitor or resistor is connected in parallel or in series, respectively, at both ends of the excitation coil [12]. The magnetic field enters a periodic state of saturation, but because the excitation signal is not pre-conditioned, it interferes with the induced signal [13]. Early research on fluxgate was purely engineering-based and lacked a quantitative analysis of the entire fluxgate process [14]. Owing to continuous fluxgate research, the quantitative analysis of fluxgate has gradually begun; however, because the core permeability is a nonlinear function during periodic saturation, fluxgate analysis treats the nonlinear problem as a linear problem, which is commonly used to equate the core hysteresis curve as trigonometric [15,16]. In [15], previous studies utilized the same excitation circuit structure and compared and analyzed that simulation without considering the actual magnetic permeability change of the magnetic core cannot achieve quantitative analysis. The waveform simulated in [15] was verified using Ansys software to demonstrate its effectiveness. However, it is difficult to analyze the performance of different core hysteresis lines quantitatively during excitation, and one can only empirically select the device parameters in the excitation circuit. This situation hinders the design and development of the entire application. In this study we considered the actual permeability curve of the core and designed an excitation circuit for a square wave excitation waveform. Further, we considered the commonly used excitation circuits for comparison. The actual permeability curve was then substituted into the excitation circuit for calculation and simulation.

The remainder of this paper is structured as follows. The Section 2 describes the principle and structure of the fluxgate and performs calculations and simulations for the examined excitation circuit. The Section 3 describes the experiments and discussion of the examined excitation circuit. The Section 4 concludes the paper.

## 2. Calculations and Simulations

**Figure 1:**
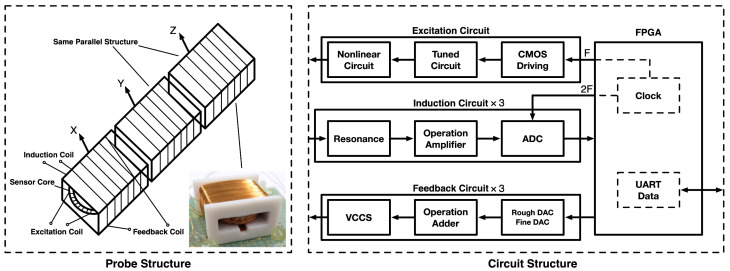
Structure of the three-axis magnetic fluxgate.

The fluxgate examined in this investigation is a three-axis digital magnetometer that uses the even harmonic method, allowing for the miniaturization of the fluxgate, improving portability, and has a wider range of possible applications. Figure 1 shows the overall structure of the magnetic fluxgate used in this study [17]. Each axis has its own excitation, induction, and feedback coils, and is arranged horizontally in parallel to form the entire three-axis probe. In addition to power supply, control, and communication circuits, the circuit section has excitation, induction, and feedback circuits for each probe coil, which together form the entire fluxgate circuit structure. In this study, the three-axis excitation coils in the probe were independent but shared the same excitation signal. According to this study, during quantitative analysis, the parameters of each coil in the three axes were considered to be mechanically consistent and strictly orthogonal. This research focused on the individual axes of the probe.

### 2.1. Excitation Circuit

**Figure 2:**
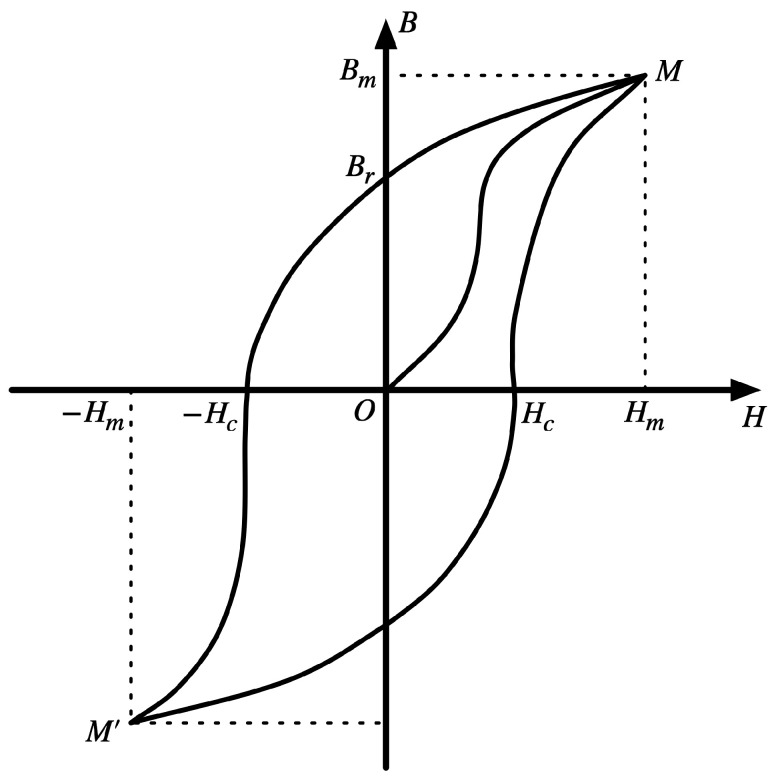
The hysteresis curve of the soft magnetic material core.

Static magnetic field measurements rely on the active generation of an excitation signal, magnetic permeability modulation of the core, and an induction signal that contains information on the magnetic field being measured [18]. The excitation circuit, which collaborates with the excitation coil to generate the excitation signal, determines the overall performance of the fluxgate [19]. The core hysteresis curve is shown in Figure 2. When the absolute value of the magnetic field at the core is less than the saturation field $H_{m}$, the magnetic induction intensity varies with the magnetic field, and the core permeability μ is approximately constant. Conversely, when the absolute value of the magnetic field at the core is greater than the saturation field $H_{m}$, the core permeability is an even function μ(t) that varies with time. According to the law of electromagnetic induction, when the excitation field $H_{exc}$ is smaller than the saturation field $H_{m}$, the measured magnetic field $H_{0}$ is not correlated with the induction signal $E_{1}$. However, when the excitation field $H_{exc}$ is larger than the saturation field $H_{m}$, the induction signal $E_{2}$ contains the measured magnetic field $H_{0}$, which is the time we regard as excitation works. Equation (1) shows the two excitation cases:

(1) $$\begin{array}{c} \{\begin{array}{ll} E_{1}=-NS\frac{dB}{dt}=-\mu \omega NSH_{exc}cos\omega t & H_{exc}<H_{m}\\ E_{2}=2\mu_{2}\omega NSH_{0}sin2\omega t & H_{exc}>H_{m} \end{array}, \end{array}$$

where $H_{exc}$ denotes the excitation magnetic field amplitude, ω denotes the excitation magnetic field frequency, $\mu_{2}$ denotes the second Fourier decomposition term of the magnetic permeability, N denotes the number of turns in the excitation coil winding, S denotes the core cross-sectional area, and B denotes the magnetic induction intensity at the probe. When the excitation is active, the magnetic field calibration of the measured magnetic field can be used to characterize the second harmonic with the largest component of the even harmonics of the induced signal.

**Figure 3:**
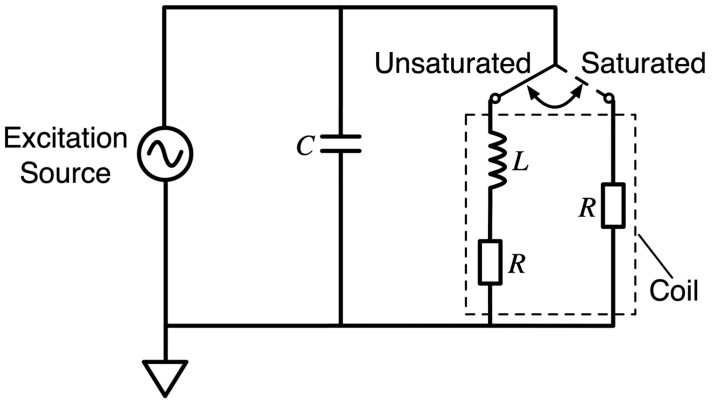
Common fluxgate excitation circuit structure.

Figure 3 shows how the fluxgate modulates the measured magnetic field into an induction signal and the common excitation circuit of a general fluxgate, where a capacitor is connected in parallel to the excitation coil and a sinusoidal signal is used as the excitation signal to drive the core with a pulse current to achieve periodic deep saturation while minimizing the power loss. This general excitation circuit is based on an ideal situation, which is unsuitable for existing miniaturized fluxgate sensors. First, pure sinusoidal signal generation is complicated, and second, the core equivalent nonlinear circuit model disregards the actual core hysteresis curve. This circuit is suitable for explaining the fluxgate–excitation principle.

**Figure 4:**
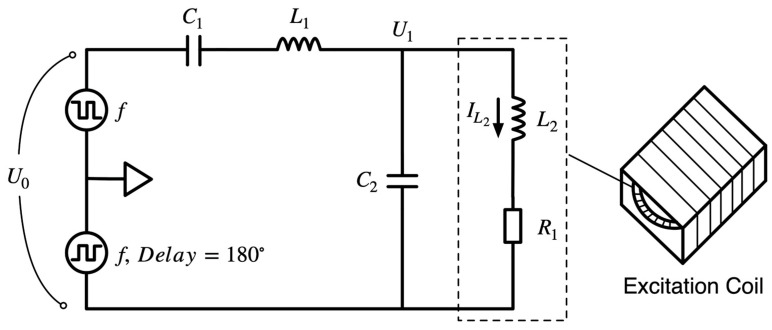
Magnetic fluxgate drive signal modulation excitation circuit structure.

The excitation circuit structure used in this study is shown in Figure 4. The solution was used for a miniature digital fluxgate and includes a power driver, signal modulation, and nonlinear circuits. The excitation signal of this circuit uses square-wave excitation that the FPGA generated, and the signal generation circuit has a simple structure and stable level suitable for direct connection to power amplifiers. Because of the power supply and external noise interference, the excitation signal is impure in the actual circuit; therefore, the signal must be tuned before transmission to the equivalent nonlinear circuit of the excitation coil. The nonlinear excitation circuit examined in this study considers the actual hysteresis curve of the core without simply equating the excitation coil to two simple circuit models, as shown in Figure 3. Because the actual core variation under periodic saturation is considered, it is more accurate than the previous conventional linearization to analyze the excitation circuit problem and can target the excitation waveform variation according to the variation of the device parameters in the circuit.

### 2.2. Mathematical Calculation

**Figure 5:**
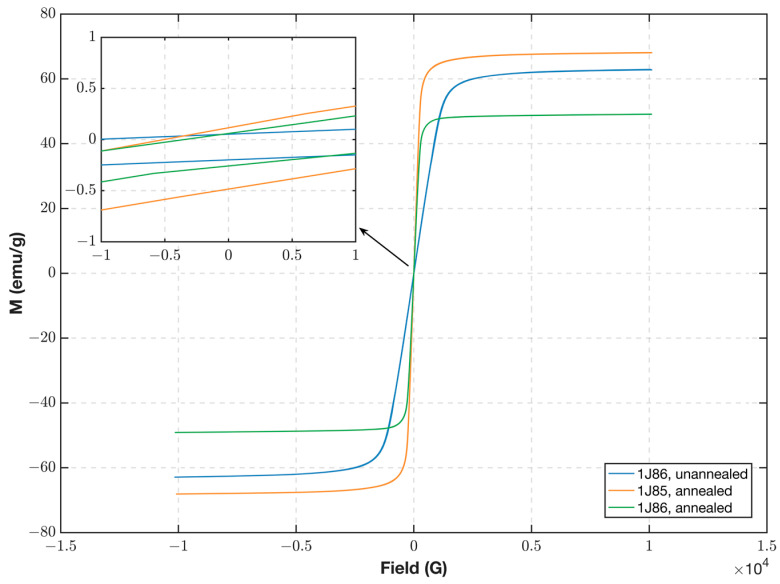
Measured hysteresis of different annealing core materials.

The hysteresis curves of the three types of permalloy soft magnetic materials were measured using a vibrating sample magnetometer at 25 °C. Figure 5 displays the findings from the measurement using a PPMS-9 (Quantum Design Company, San Diego, CA, USA) device. The pre- and post-annealing magnetic properties of both materials are clear. The fluxgate typically uses annealed magnetic material as the core because the magnetic permeability of the core after annealing is significantly larger than that of the unannealed core, and the larger the permeability of the core, the easier it is for it to enter deep saturation under the same excitation field to obtain a better excitation effect. The difference in the magnetic properties between different grades of cores with the same annealing treatment is also clear. The saturation field strength of 1J86 annealed is less than that of 1J85 annealed, whereas the remanence of 1J86 annealed is less than that of 1J85 annealed. Additionally, 1J86 annealed shows better magnetic properties for the fluxgate. The 1J86 annealing was used as the core material for the experiments.

**Figure 6:**
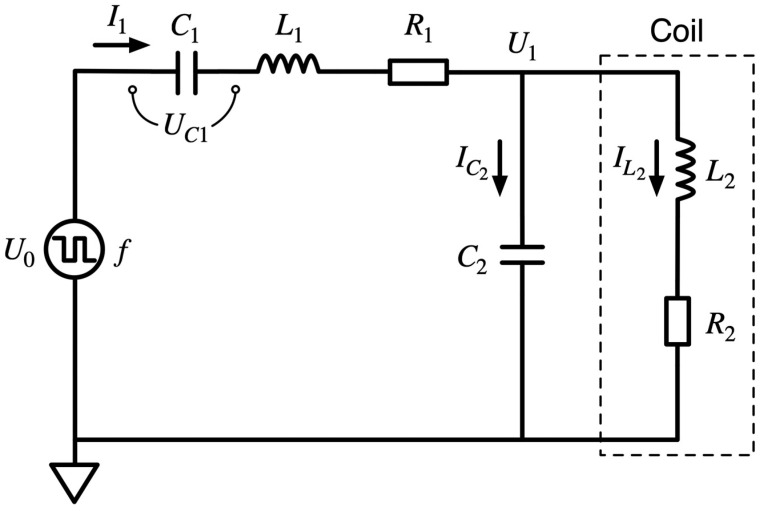
Mathematical calculation of the equivalent excitation circuit structure.

The excitation circuit examined in this study is shown in Figure 4. For mathematical analysis, Figure 6 is based on Figure 4 and considers the actual permeability changes. The actual resistance and capacitance of each component in the circuit were considered and marked with the current direction to facilitate the development of mathematical models and analysis. In this research, the excitation voltage frequency f is set at 9.6 kHz because the equivalent impedance of the probe excitation coil used at this frequency is the smallest and the maximum excitation power can be obtained at this frequency. According to the excitation circuit shown in Figure 6, a set of differential equations can be written according to the relationship between the voltage and current in the circuit in Equation (2):

(2) $$\begin{array}{c} \{\begin{array}{l} L_{2}\frac{dI_{L2}}{dt}=U_{1}-I_{L2}R_{2}\\ L_{1}\frac{dI_{1}}{dt}=U_{0}-U_{C1}-U_{1}-I_{1}R_{1}\\ C_{2}\frac{dU_{1}}{dt}=I_{1}-I_{L2}\\ C_{1}\frac{dU_{C1}}{dt}=I_{1} \end{array}. \end{array}$$

The equivalent inductance $L_{2}$ of the excitation coil in Equation (2) is not constant. The inductance varies with the excitation field; it is higher when the excitation field is not saturated and significantly lower when it is. The excitation field $H_{exc}$ size can be obtained according to the ampere-loop theorem, as follows:

(3) $$\begin{array}{c} H_{exc}=\frac{I_{3}N}{l}. \end{array}$$

According to the excitation coil inductance equation,

(4) $$\begin{array}{c} L=\frac{N^{2}S}{l}\frac{dB}{dH_{m}}, \end{array}$$

where l denotes the circumference of the toroidal core, the differential permeability of the core can be derived from the measured hysteresis curve of the core, and the corresponding relationship between the equivalent inductance of the probe and the change in current can be determined by solving the system of differential equations (Equation (4)). The specific trends of the voltage and current at each node of the circuit during the excitation process can be determined using the fourth-order Runge–Kutta method.

**Figure 7:**
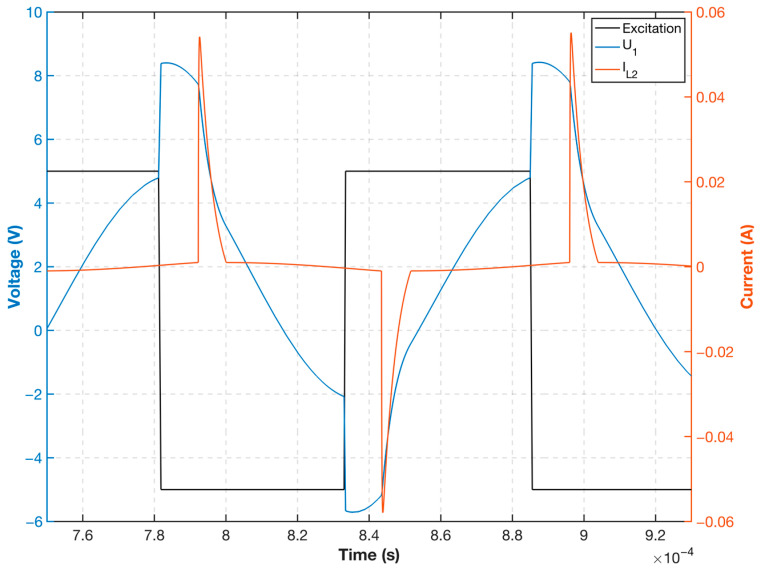
Excitation voltage and probe current mathematical calculation waveform.

Based on the measured core hysteresis curve and the actual circuit device parameter calculation, the voltage and current waveforms of the equivalent inductance were plotted, as shown in Figure 7. This finding demonstrates that when the excitation voltage level is switched, the parallel capacitor discharges; when the excitation field generated by the current is greater than the core saturation field $H_{m}$, the probe equivalent inductance $L_{2}$ decreases rapidly as the permeability decreases, and the resistance to current changes weakens after the inductance decreases significantly. Thus, the probe excitation current will appear as a pulse spike, rapidly accelerating the entry of the core into deep saturation. Subsequently, the current decreases, and when it is smaller than the core saturation field, $H_{m}$, the equivalent probe inductance returns to a larger inductance value. The current change decreases until the next excitation voltage level is converted to maintain the smaller current value to achieve the deep saturation effect of small power.

### 2.3. Nonlinear Simulation

Most of the fluxgate excitation circuit research is based on the mathematical analysis method described above, in which the calculation of the equivalent inductance of the probe relies on the measured hysteresis curve data of the core, and the magnetic properties of the probe excitation coil after winding differ from the measured hysteresis curve of the core because the core hysteresis curve measurement is measured only by sampling the core material. Simultaneously, the models for resistance, capacitance, and inductance are ideal linear models owing to the mathematical circuit model. The excitation circuit simulation using the SPICE software LTspice can solve the problem of the model singularity of resistance, capacitance, and inductance. For the probe-equivalent nonlinear inductor, this study uses the CHAN nonlinear transformer model, which considers the nonlinear relationship between the winding current on the core [20]. In LTspice software, the same circuit as Figure 4 was set up using the built-in nonlinearity CHAN model of the simulation software. With the actual device and coil parameters, the voltage and current waveforms at each node in the circuit were simulated and solved. Based on the waveforms, the coil and excitation circuit parameters were optimized to achieve the best excitation effect of the fluxgate. In this model, the magnetic field is calculated using Equation (5), as follows:

(5) $$\begin{array}{c} H_{eq}=\overset{n}{\underset{i=1}{\sum }}\frac{k_{i}N_{i}I_{i}}{l_{mag}}, \end{array}$$

where $k_{i}$ denotes the coefficient of the winding-core coupling, $N_{i}$ denotes the number of turns of winding i, $I_{i}$ denotes the magnitude of the current flowing through winding i, and $l_{mag}$ denotes the effective magnetic circuit length of the core. Additionally, the relationships between the magnetic field strength, magnetic field, magnetic permeability, magnetic flux, inductance, and current were obtained.

(6) $$\begin{array}{c} \{\begin{array}{c} B=\mu_{0}\mu_{r}H=\frac{\phi }{S}\\ \phi =LI \end{array}, \end{array}$$

where $\mu_{0}$ denotes the magnetic permeability in a vacuum, $\mu_{r}$ denotes the average relative permeability of the core, and ϕ denotes the magnetic flux. The coupling effect of the winding and core in this nonlinear transformer model can be considered to determine the equivalent inductance $L_{eq}$:

(7) $$\begin{array}{c} L_{eq}=\frac{\mu_{0}\mu_{r}H_{eq}NS}{l}. \end{array}$$

The inductance based on this model is closer to the actual probe-equivalent inductance variation than Equation (4) when the winding coupling is considered. In addition to being nonlinear equations, the magnetic induction strength B and magnetic field strength H depend on the magnitude of the historical magnetic field on the core. The model considers the effect of the historical magnetic field on the change in permeability under the Newton–Raphson algorithm, as well as the effect of frequency on the coercivity $H_{c}$ and winding parasitic resistance $R_{w}.$ The validity of the model was verified through simulation. Therefore, the model analysis presented above is comprehensive and suitable for both simulating and analyzing the fluxgate nonlinear excitation circuits.

The model was modeled in a circuit simulation based on the circuit shown in Figure 4, where the nonlinear CHAN model can set the core magnetic property parameters of coercivity, saturation magnetic induction strength, and residual magnetic field, as well as the probe mechanical parameters of magnetic length, cross-sectional area, number of coil turns, and core air gap. The model parameters were set according to actual probe conditions. In the simulation, the equivalent inductance voltage integral of the probe can be used to characterize the magnetic induction strength, whereas the equivalent inductance current can be used to characterize the magnetic field strength. Then, the hysteresis curve expression of the model in the simulation can be calculated using the structural parameters of the turns and the cross-sectional area of the probe:

(8) $$\begin{array}{c} \;f\left(B,H\right)=\frac{\int_{0}^{t}U\left(L_{2}\right)dt}{N^{2}SI\left(L_{2}\right)}.\; \end{array}$$

**Figure 8:**
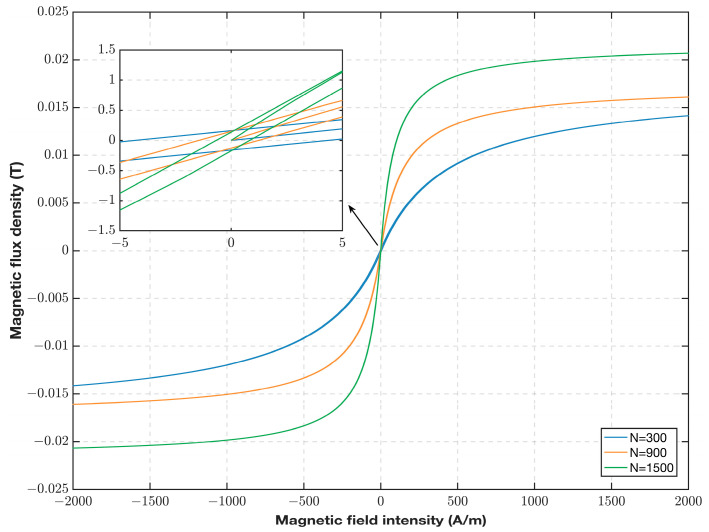
Hysteresis curves for different number of turns of the CHAN model.

Figure 8 shows the hysteresis curve of the model calculated using Equation (8). According to Figure 8, the hysteresis curve of the model matches the trend of the actual hysteresis curve, and it changes as the core magnetic performance parameters and probe structure parameters of the model change. When the remanence, coercive force, and number of turns of the winding changed, the hysteresis return area also changed. When the number of turns is small, the overall permeability of the coil is small; however, when the number of turns is large, the overall saturation magnetic induction intensity of the coil is large. This characteristic can be seen by analyzing the change in the model hysteresis curve after the change in parameters and considering the requirement of the fluxgate sensor to enter deep saturation quickly. The change in the hysteresis curve owing to the change in the number of turns of the coil is consistent with the actual situation, and the model parameters can be selected according to the actual probe situation to achieve the optimal excitation effect.

**Figure 9:**
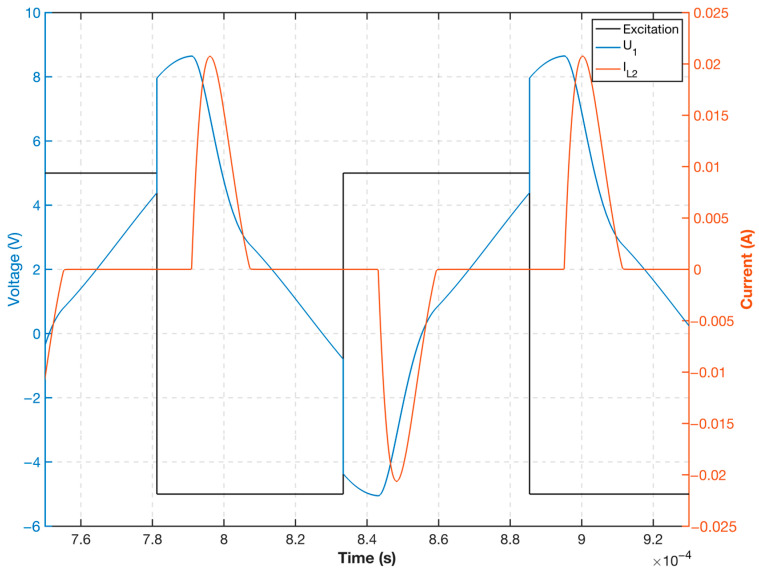
Excitation circuit simulation voltage and current waveforms.

Figure 9 displays the voltage and current waveforms for the selected CHAN model in accordance with the actual design of the simulation circuit and by choosing the same device parameters and excitation signal frequency as in the mathematical calculation. The voltage and current waveforms obtained using the CHAN nonlinear model are consistent with the mathematical calculations in the previous section and both can accurately depict the voltage and current variations of the nonlinear excitation circuit during the entire excitation period. Both mathematical modeling and simulation modeling of the fluxgate excitation circuit can better analyze the changes in the corresponding excitation waveform following an excitation change during the excitation process; however, the mathematical modeling analysis overly relies on the actual core measurement data, and the core hysteresis curve measurement data disregard the actual winding nonlinear coupling situation. Therefore, the nonlinear model circuit simulation considers the above problems. Simultaneously, the nonlinear excitation circuit simulation is more suitable for nonlinear analysis of the actual fluxgate excitation circuit because the probe parameters are flexible.

## 3. Experiments and Discussion

**Figure 10:**
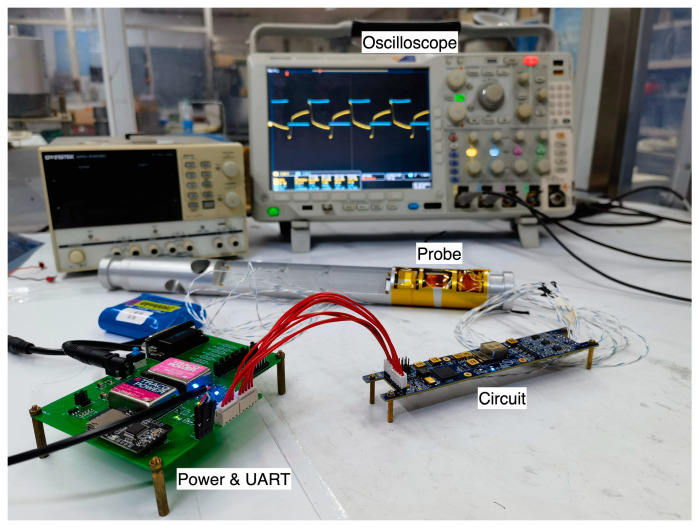
Excitation circuit waveform measurement experiment.

Figure 10 illustrates the experimental verification that was performed to confirm the feasibility of the analysis of the nonlinear fluxgate sensor excitation circuit in this study. The experimental equipment includes an oscilloscope, a self-research probe, a miniaturized downhole self-research fluxgate circuit, a power supply, a communication isolation and adapter board, and a battery power supply to reduce the power supply noise. The power supply and communication adapter board, which simulate the power supply of the external downhole equipment, are used only during experiments to facilitate signal testing and debugging.

**Figure 11:**
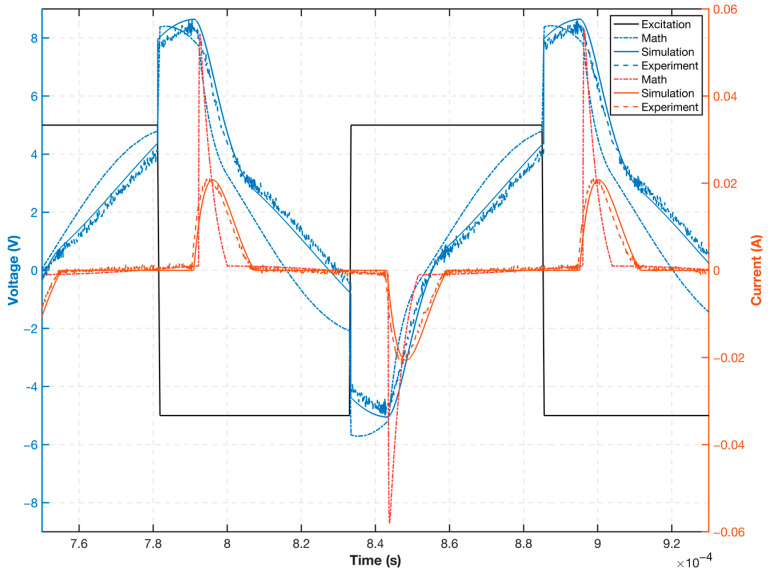
Mathematical, simulated, and experimental excitation circuit voltage and current waveforms.

The circuit used in the experiment was consistent with that in the mathematical and simulation analyses, and the data for all three in the same configuration are plotted as shown in Figure 11. Note that the circuit and the excitation voltage $U_{0}$ used are both consistent. Although they can both reflect the equivalent excitation voltage $U_{1}$ and the equivalent inductance current $I_{L2}$ changes caused by core saturation in the excitation circuit, the simulated waveform is significantly more similar to the experimental waveform than the mathematical analysis waveform. Particularly, the magnitude of the equivalent inductor current $I_{L2}$ calculated using the mathematical model is larger than that of the simulation and experimental results, and the phase of the current spike after saturation does not reflect the phase lag in the real case, whereas the excitation current calculated using the mathematical model has a faster current jump after saturation. For voltage $U_{1}$, the experimental results are consistent with the simulated results, and both voltage phases are consistent with the jump in the excitation voltage $U_{0}$ voltage switch. The voltage remains constant for a period corresponding to the current waveform before entering saturation, and after the current is reduced, the simulated and experimental voltage waveforms converge.

**Figure 12:**
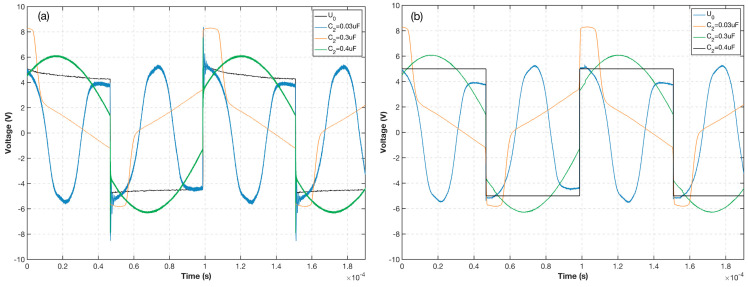
Comparison of excitation waveforms with different capacitors. (**a**) Experiment. (**b**) Simulation.

To further analyze and compare the correlation between the simulation and experimental results, the capacitance value $C_{2}$ of the shunt connection at both ends of the probe was changed under the same circuit topology to observe the effect of resonant capacitance on the saturation of the probe core. In the experiment, the corresponding capacitance values for the core in different saturation states were obtained by adjusting the capacitance value of the shunt capacitor and observing the probe excitation voltage $U_{1}$ waveform. Figure 12 displays the simulation and experimental results using the corresponding capacitance values from the circuit simulation. Note that the base value is the capacitance value of 0.3 uF corresponding to the core entering deep saturation normally. The base value is then appropriately adjusted upward and downward to 0.4 uF and 0.03 uF, respectively, and the adjusted experimental waveform is consistent with the simulated waveform in terms of overall trend and phase. The larger the absolute value of the capacitance value adjusted on the base value, the farther the core is from deep saturation, which causes the permeability to remain constant for a while during the excitation cycle and affects the normal coupling between the measured magnetic field and the induced voltage. When the capacitance value increases, the voltage waveform is reflected as a flatter waveform. Conversely, when the capacitance value decreases, the voltage waveform changes phase several times during the half-cycle. Both of these conditions prevent the core from completely entering deep saturation, which eventually affects the overall performance of the flux gate. Therefore, it is possible to calculate the optimal circuit parameters through circuit simulation, which accelerates the overall device development and research on the influence of the excitation component on the overall fluxgate performance.

**Figure 13:**
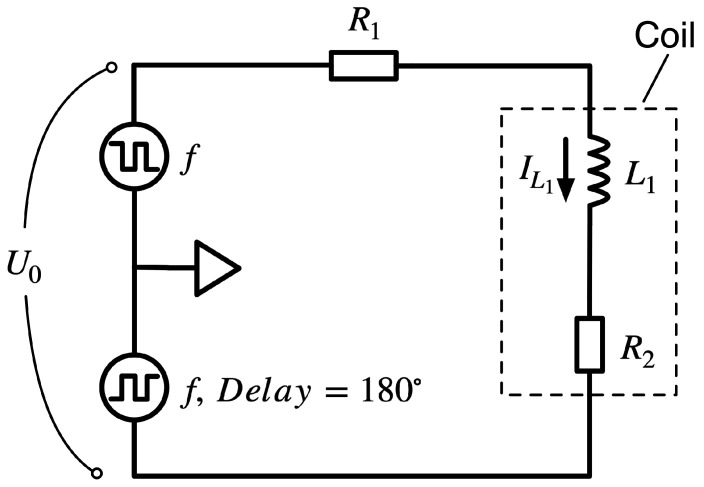
Single resistor excitation circuit structure.

After studying and verifying the consistency of the simulation and experimental results on the excitation circuit structure used in this study, a comparative simulation and experimental analysis of the excitation circuit structure in Figure 13 was performed to verify the usability of the CHAN nonlinear model under different fluxgate excitation circuits. Figure 13 shows the most basic fluxgate excitation circuit. This scheme only requires adjusting the resistance value of the component in the circuit to achieve deep saturation of the excitation current during the excitation period. However, the excitation power consumption of this scheme is large and is not suitable for miniaturized fluxgate application scenarios.

**Figure 14:**
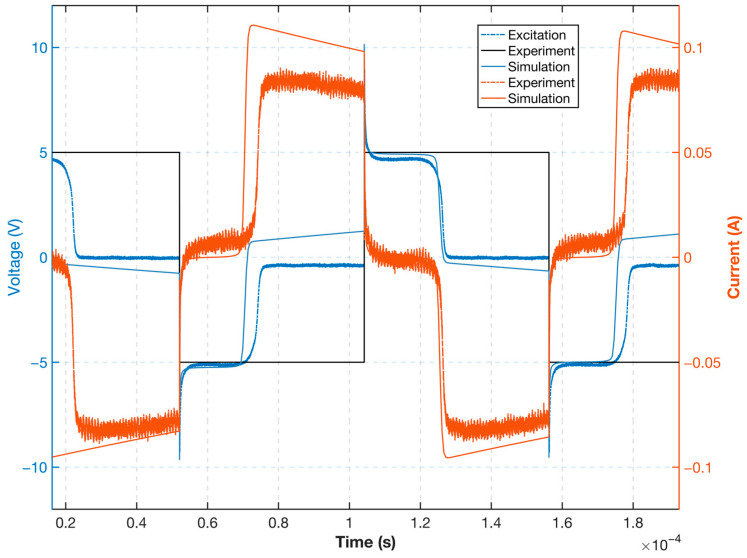
Comparison of simulated and experimental waveforms of a single resistor excitation circuit.

The simulation and experimental comparison waveforms of the excitation voltage and current for the selected optimal resistor resistance are shown in Figure 14. Note that the untuned signal in the circuit results in trends that are essentially the same for both, while the noise in the actual experimental circuit causes numerical differences between the simulation and experimental results. The probe core enters deep saturation under optimal resistance, while the excitation current is controlled within a certain range to ensure the overall excitation efficiency and performance. The excitation waveforms corresponding to different resistance values under this excitation circuit can be simulated by modifying the resistance value in the simulation, which allows for the quick selection of the best circuit device parameters.

## 4. Conclusions

This paper summarizes decades of research on the nonlinear analysis of fluxgate excitation circuits. The common method is to equate the hysteresis curve of the fluxgate core to a linear model or to an inverse tangent function before performing mathematical modeling calculations using this method. Finally, the relationship between excitation waveform variation and the fluxgate excitation circuit was analyzed throughout the excitation period. Through a detailed theoretical analysis of the fluxgate excitation circuit, this method was found to be more suitable for the qualitative analysis of the excitation circuit rather than the analysis of the specific waveform phase and trend of the fluxgate excitation circuit. In all cases, the error between the simulated current of the excitation circuit and the measured current was within 5%.

In this study, for fluxgate nonlinear excitation circuits, a mathematical model of the excitation circuits was devised to improve the accuracy of the analysis using the core real measurement hysteresis curve. Further, the simulation accuracy of excitation waveforms was improved using the CHAN model, which considers the nonlinear coupling between the core and the winding when simulating the excitation circuit. In the excitation circuit waveform analysis, the advantages and shortcomings of using the core-measured data in the mathematical model were examined, and the enhancement effect of using the nonlinear probe equivalent model in the simulation of the probe history coupling and the analysis efficiency were analyzed. The feasibility of both in the nonlinear analysis of fluxgate excitation circuits was then verified by comparing the mathematical modeling and simulation analysis in the experiments, with the simulation and experimental results typically being more consistent. Subsequently, several sets of experiments were conducted to verify that the simulation of the CHAN nonlinear model can accurately analyze the fluxgate excitation circuit voltage and current waveforms under different circuits and device parameters, and it can assist in verifying the circuit structure and device parameters and significantly improve the efficiency of the actual fluxgate development.

## Data Availability

Not applicable.
